# The effects of morphine abuse on sperm parameters, chromatin integrity and apoptosis in men

**DOI:** 10.5935/1518-0557.20210110

**Published:** 2022

**Authors:** Saeed Ghasemi-Esmailabad, Amir Hossein Talebi, Ali Reza Talebi, Sepide Amiri, Mojgan Moshrefi, Majid Pourentezari

**Affiliations:** 1 Research and Clinical Center for Infertility, Yazd Reproductive Science Institute, Shahid Sadoughi University of Medical Sciences, Yazd, Iran; 2 Abortion Research Center, Reproductive Sciences Institute, Shahid Sadoughi University of Medical Sciences, Yazd, Iran; 3 Department of English language and literature, Faculty of humanities and social sciences, Yazd university, Yazd, Iran; 4 Andrology research center, Reproductive Sciences Institute, Shahid Sadoughi University of medical sciences, Yazd, Iran; 5 Department of Biology and Anatomical Sciences, Faculty of Medicine, Shahid Sadoughi University of Medical Sciences, Yazd, Iran; 6 Medical Nanotechnology & Tissue Engineering Research Center, Yazd Reproductive Science Institute, Shahid Sadoughi University of Medical Sciences, Yazd, Iran

**Keywords:** morphine, sperm parameters, chromatin, DNA, human

## Abstract

**Objective:**

Morphine is one of the major psychoactive chemicals in opium that can increase the production of free radicals and thus can negatively affect spermatogenesis. The purpose of this study was to demonstrate the effects of morphine consumption on sperm parameters, DNA integrity and apoptosis in men taking morphine.

**Methods:**

In this case-control study, 30 man abusing morphine (cases) and 30 healthy men (controls) were compared in sperm parameters (count, motility and morphology) and sperm chromatin quality, with Aniline Blue (AB), Toluidine blue (TB) and Chromomycin A3 (CMA3) stains. The participants were matched for age, weight, amount and duration of cigarette smoking.

**Results:**

In men with morphine dependency, sperm progressive and total motility (*p*=0.038 and *p*=0.000, respectively) showed a significant decrease compared to the control group. Concerning morphine abuse, although morphine can decrease the sperm chromatin condensation and increases the rate of sperm apoptosis, these differences were not statistically significant.

**Conclusions:**

According to our results morphine dependence can reduce male fertility by affecting sperm parameters.

## INTRODUCTION

Infertility is one of the major problems among young couples, and one out of every six couples suffers from infertility. Although the problem of infertility is multifactorial, it has been shown that jobs, toxicants, and the environment are very important issues (Gannon & Walsh, 2015; Cariati *et al*., 2019). Morphine is the most well-known and oldest type of drug that addiction to it may cause serious problems to health, family, and social communities. Increasing the rate of crime and community violence, low productivity and political instability are some of the results of morphine abuse (Singer, 2008). Opium is extracted from the pod of the opium poppy seed (*Papaver somniferum*). They contain opium alkaloids such as morphine as well as other synthetic derivatives such as codeine and heroin. They can attach to three main receptor classes - mu (µ), delta (δ) and kappa (κ) - dependent of the supergroup G protein receptors, which respond to three main groups of endogenous drugs - peptides (Endorphins, enkephalins and dinorphins) (Vuong *et al*., 2010). Endogenous opiates are physiologically implicated in several body functions, including motor function, various components of the immune system, gastrointestinal function, cardiovascular system, nerves and glands, cognitive system and pain control. Opioid analgesics, such as oxycodone, propoxifene, hydrocodone, fentanyl, and methadone, are often advised for musculoskeletal disorders and rheumatism, which provide long-term pain relief. However, the potential for addiction is obvious (Vuong *et al*., 2010). Opioid receptor antagonists, such as naloxone and naltrexone, are used to reverse the effects of opioids. The inhibitory effects of opioids on testosterone function are widely known. However, its effects on spermatogenesis are controversial (Ahmadnia *et al*., 2016). Opiates affect the pituitary system, sex organs, and sexual functions (Baharin *et al*., 2020). Regular use of morphine, especially in patients using morphine sulfate vials medically for relieving pain, can decrease the number of sperms and cause sperm deformity and dysfunction in men (Takzare *et al*., 2016). Like other opiates, morphine exerts its effect through opioid receptors (Baharin *et al*., 2020). These receptors are dispersed in the limbic system, amygdala, hippocampus, thalamus, and hypothalamus and they exist in the testes too (Vicente-Carrillo *et al*., 2016). Opioid abuse is a chronic recurrent disorder. Consecutive opioid use increases the production of reactive oxygen species (ROS), reduces antioxidant capacity and the number of synapses, and ultimately contributes to opioid dependence (Gipson *et al*., 2014; Mameli & Lüscher, 2011). ROS regulates inflammatory cytokines as well as it increases matrix metalloproteinase (MMP) activity (Salarian *et al*., 2018). According to previous studies, treatment with morphine activates MMP-9, which may help cause morphine tolerance (Nakamoto *et al*., 2012). These free radicals or ROS cause damage to the cell membrane and DNA fragmentation (Ribas-Maynou *et al*., 2020).

Assessment of sperm nuclear chromatin is important in male fertility research. Throughout spermatogenesis, sperm chromatin is compacted more and more because of histone replacement at first by testis-specific nuclear proteins, afterwards it happens via transitional proteins and eventually by protamine's (Talebi *et al*., 2012). Disulfide bonds between protamine molecules are important for sperm density and nuclear stabilization. This nature of nuclear density is believed to assert the sperm genome from damage such as oxidative stress, high temperatures, and acid-induced DNA denaturation (Carrell *et al*., 2007). Having sperm with immature chromatin and markers of apoptosis may be a cause of infertility (Dehghanpour *et al*., 2020). So due to the importance of sperm parameters and the role of sperm chromatin/DNA integrity in fertility and the probable deleterious effects of morphine on it, the purpose of this study was to investigate the impact of opiate usage on sperm fertility potential in opium-addicted males.

## MATERIALS AND METHODS

The case group were men (n=30 and age, 20-40 years) consuming morphine (1g of morphine per day) for at least one year of consumption. They had no history of surgery, metabolic and infectious diseases, endocrine abnormalities and taking drugs and alcohol that may affect the male reproductive system and fertility. They also didn't have any exposure to toxic substances for a long time. The control group (n=30) was chosen between men from couples referred to Yazd Infertility Research Institute, considered fertile men with normal sperm parameters. They didn't have any history of drug-abuse, alcohol consumption or cigarette smoking. Before entering the study, the participant was explained about the study and a written consent was obtained. The project was approved by the Ethical Board of the Yazd Infertility Research Institute (IR.SSU.RSI.REC.1396.22).

### Sperm preparation

Semen samples were acquired by masturbation under standard protocol after sexual abstinence from 2-4 days. Each sample was placed in 37°C for 1 hour and then, it was examined for motility, number, and morphology (Talebi *et al*., 2014). Multiple smears were also taken from patients for sperm chromatin/DNA assessment (Talebi *et al*., 2014).

### Sperm chromatin and DNA integrity tests

To evaluate sperm chromatin/DNA integrity, four experiments were carried out: the terminal deoxynucleotidyl transferase dUTP nick end labeling (TUNEL) assay for apoptosis or DNA fragmentation; the Chromomycin A3 (CMA3) for sperm protamine deficiency; the Aniline Blue (AB) staining for detection of excessive histones in the process of chromatin condensation; and the Toluidine Blue (TB) for sperm chromatin compaction status (Talebi *et al*., 2014).

### Chromomycin A3 (CMA3) staining

The percentage of sperm protamine deficiency was examined by the CMA3 staining previously reported by Pourentezari *et al*., the semen sample was fixed in Carnoy solution for 10 minutes at 48°C and a smear was prepared from it. Each slide was stained with 100 µl of CMA3 solution (Sigma) (0.25 mg/ml Mcllvain Buffer containing 10 mM magnesium chloride at pH=7) for 20 minutes to remove residues. The excess dye washes the slides in PBS solution and examines them with a 100x magnification under fluorescence microscopy. About 200 sperm were tested in each sample. Heads of sperm that are deficient in protamine are bright yellow (CMA3^+^), which is reported as the percentage of CMA3^+^ sperms, and those that are naturally occurring are light yellow (CMA3^-^), and as the percentage of CMA3^-^ sperm is reported (Talebi *et al*., 2018).

### Aniline blue (AB) staining

AB staining was used to evaluate the chromatin density of the sperm samples. In this method, prepared and dried smears were stabilized in air temperature using 4% formalin. They were then washed with water and stained in 5% AB in 4% acetic acid solution (pH 3.5). Each fixation and staining step were performed at room temperature for 5 minutes. The slides were washed with water, dried at room temperature, and evaluated using a light microscope. At least 200 sperm were counted and examined. Dark-stained sperm were considered immature sperm with excess histone and abnormal chromatin (Talebi *et al*., 2018).

### TB staining

TB staining was performed as described in our previous study. To evaluate the status of chromatin, a thin smear was prepared on the slide. The smears were dried in air temperature and fixed with 96% ethanol-acetone (1: 1) at 4°C for one hour. The slides were then placed in 0.1 NHCl at 4°C for 5 minutes, followed by washing 3 times with distilled water for 2 minutes. In the next step for staining with TB, 0.005% were exposed to room temperature for 5 minutes. TB dye was used to assess the chromatin. The head of the sperm is light blue with healthy chromatin and those with fragmented or abnormal chromatin are dark purple. A total of 200 sperm per slide was observed and evaluated using a light microscope (Talebi *et al*., 2013; 2018; Pourentezari *et al*., 2016).

### TUNEL assay

The extent of DNA damage was assessed using the TUNEL method, previously described by Talebi *et al*., which is as follows: first, the semen was washed with PBS solution and stained by preparing a smear on the slide according to the instructions of the TUNEL kit. According to this method, the fluorescent dye is attached to the ends of broken DNA fragments by the rTdT enzyme, and fluorescein-12-dUTP marks the DNA. Examination of the fluorescent green by a 100-magnitude fluorescence microscope (BX51; Olympus; Japan) in the posterior region of the sperm head indicates TUNEL^+^ (DNA-damaged sperm) and red indicates TUNEL^-^ sperm (DNA sperm is healthy) which is reported as a percentage. About 200 sperm are tested for each sample (Talebi *et al*., 2013).

### Statistical analysis

We used the SPSS ver. 20.0 (IBM Corp., Armonk, NY, USA) for all data analysis. The data were expressed as mean ± standard deviation. We used the student t-test to evaluate the data, and the term "statistically significant" was used to show *p*<0.05 for sperm parameters and chemical tests.

## RESULTS

Table 1 shows the means and statistical analyses of the sperm count and motility in the two groups. Regarding sperm progressive motility and total motility, we saw significant differences between the two groups (*p*=0.038 and *p*=0.000, respectively), but the results of sperm count didn't show any statistically significant differences between control and case groups.

**Table 1.** The results of semen analysis in two groups.

Variables	Control Mean±SD	Case Mean±SD	*p*-value
Count (10^6^cell/ml)	105.7±59.24	73.76±45.95	0.297
Progressive (%)	58.3±10.93	35.76±15.95	0.038
Non progressive (%)	13.06±4.63	10.36±3.39	0.067
Total Motility (%)	71.36±8.38	46.13±17.5	0.000

Student’s t-test, *p*<0.05.

**Figure 1:**
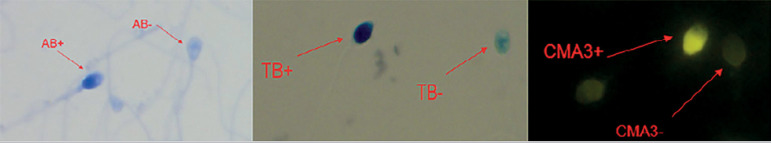
AB, TB and CMA3 staining, ×100 eyepiece magnification.

Table 2 shows the results of the analysis of sperm chromatin quality and sperm apoptosis. This table reveals that although the percentages of spermatozoa in AB, TB, CMA3 staining, and TUNEL test were higher among the cases than in controls, these differences were not statistically significant. In other words, abnormal sperm chromatin packaging and apoptosis may increase with opium abuse, but this increase is not significant (Figure 1).

**Table 2.** The results of sperm DNA evaluation in two groups.

Variables	Control Mean±SD	Case Mean±SD	*p*-value
Aniline Blue (%)	29.1±12.17	40.6±12.98	0.688
Toluidine Blue (%)	30.2±11.07	49.3±9.63	0.525
CMA3 (%)	22.8±8.88	30.8±7.53	0.924
TUNEL (%)	9.03±2.94	10.26±2.95	0.820

Student’s t-test, *p*<0.05.

## DISCUSSION

Infertility affects 15% of couples of childbearing age worldwide (Sun *et al*., 2019). This problem is seen in 8% of married couples in Iran (Safarinejad, 2008). In recent years, there has been a decrease in male fertility and an increase in abnormal semen parameters. It is believed that one of the main reasons for the increase in semen parameters is the increased exposure to toxic substances in the environment. These causes may include chemicals, radiation, and drug abuse (Safarinejad, 2008). Illegal drugs are reported to adversely influence male fertility. These include marijuana, methamphetamines, cocaine, opioids, and anabolic-androgenic steroids (Safarinejad, 2008). Substance abuse is a growing social and medical problem, both in developed and developing countries. In general, studies on the effects of morphine on sperm parameters and the male reproductive system can be divided into two parts. Part of these studies was carried out in animals, and the other part in humans. Animal studies have shown that rats exposed to morphine are apparently capable of mating, but this mating does not lead to a favorable pregnancy. These findings suggest that opioid abuse has led to defects in their mating behaviors, as well as imperfect fertilization and pregnancy. It also has an adverse effect on fetal development and subsequent development (Cicero *et al*., 2002).

According to the results of this study, the use of morphine reduces sperm parameters, especially motility, and reduces the quality of chromatin. Opioid systems can lead to completely different and paradoxical effects on sperm count and motility (Vicente-Carrillo *et al*., 2016; Wang *et al*., 2017; Shuey *et al*., 2008), sperm modification (Albrizio *et al*., 2006; 2010), and other reproductive factors depending on opioid receptor subtypes, affinity, expression/localization patterns and opiate concentration level (Subirán *et al*., 2011). In our study, the number of sperm in the men in the morphine dependency group decreased compared to the control group, but this decrease was not significant. Based on studies of opioid peptides that could influence cell proliferation and apoptosis, Morphine can also augment testis cell apoptosis (Li *et al*., 2004). However, anti-apoptotic effects of morphine have also been reported, mainly through activation of delta-opioid receptors (Tang *et al*., 2011). Since preserving the delicate balance between cell survival and death is of extreme importance for the proper development of testicular cells and subsequent fertility, a better understanding of the concept of the opioid system in these processes may help explain the role of opioid peptides on testicular homeostasis and even etiology of many infertility instances in patients taking opioid medications and in morphine abusers.

Opioids, whether produced endogenously or exogenously, are capable of binding to opioid receptors in the hypothalamus, pituitary, and even the testis, and influence the function of the sexual glands (Katz & Mazer, 2009). A decrease in sex hormone levels or interference in the pulsatory secretion of GnRH hormone at the hypothalamus level and the resultant reduction in the secretion of FSH and LH hormones from the pituitary gland are some known effects of opioids on the reproduction system (Ahmadnia *et al*., 2016). Morphine administration has been shown to detract catalase levels and SOD activity, according to studies. Superoxide dismutase (SOD) acts as the first line of antioxidant protection against ROS (Salarian *et al*., 2018).

Safarinejad *et al*. (2013) in a study on human society concluded that there is a correlation between morphine use and decreased sperm parameters. They also observed that chromatin damage and DNA fragmentation index increased in patients consuming morphine. In the present study, the quality of chromatin was reduced in the men with morphine dependency, but it was not significant compared to the control group. In addition, sperm motility and morphology were decreased in these individuals; in our study, a decrease in motility was also observed, but we did not examine sperm morphology. This negative effect on blood hormone levels was also evident, as testosterone levels and spermatogenesis decreased, but they reported no change in FSH and LH levels (Safarinejad *et al*., 2013).

Numerous studies have shown that morphine increases blood nitric oxide, Morphine can increase NO production by regulating intracellular calcium and activating calcium/calmodulin-dependent nitric oxide synthase (Stefano *et al*., 1996). Studies have also demonstrated a correlation between nitric oxide and sperm acrosome and tail in mice and humans. It seems that nitric oxide can have reducing effects on sperm motility by decreasing ATP levels (Weinberg *et al*., 1995). Nitric oxide can impair sperm mitochondrial membrane, thereby releasing C chromosome, causing caspase cascade activity and stimulating apoptosis (Jalili *et al*., 2016).

In the present study, the rate of sperm apoptosis, which was examined by the tunnel test, increased in the morphine group compared to the control group, but this increase was not significant. Apoptosis is indicated as an important mechanism, by which opioids may be involved in testicular homeostasis or enhance toxicity to testicular germ cells and somatic cells. Although there are many extensive studies about morphine and apoptosis, the potential effect of morphine to induce cell death by apoptosis is a controversial issue. Long time exposure to mu opioid receptor agonists has been reported to induce proapoptotic effects on various types of human and animal normal and cancer cell lines. Accordingly, morphine enhances apoptosis in tissue and cells through up-regulation of various proapoptotic proteins, nitric oxide and ROS pathways (Soltanineghad *et al*., 2019).

The opioid system is the key mediator in cell biological communication, containing receptors for endogenous and exogenous opioid peptides. Opioid peptides exert their physiological actions at both central and peripheral levels, through 3 main classes of opioid receptors: the mu-opioid receptor (MOR), the delta-opioid receptor (DOR) and the kappa-opioid receptor (KOR), which are distributed in various organs and tissues (Soltanineghad *et al*., 2019). Evidence for the widespread presence of opioid peptides and receptors in various tissues of the male reproductive system suggests that opioids may play a role in regulating reproductive function. The opioid system can participate in the regulation of the male reproductive system at multiple levels, including the levels of the central nervous system (particularly through the hypothalamic-pituitary-gonadal axis), the testes level and sperm level (Subirán *et al*., 2011). The presence of mu, delta and kappa opioid receptors on human and rodent sperm and Sertoli cells has been previously reported (Jenab & Morris, 2000). Despite the role of opioid peptides in regulating testicular function, long-term morphine use is associated with several reproductive complications, that put users at venture for both infertility and hypogonadism (Khademi *et al*., 2016; Brennan, 2013; Daniell, 2002). The direct and indirect effects of opioid peptides on the testes, including a decreased production of sperm, testicular interstitial fluid, testosterone and other sex hormones, demonstrated that the opioid system might contribute to male reproductive dysfunction (Khademi *et al*., 2016; Brennan, 2013; Moradi *et al*., 2015).

## CONCLUSION

According to the present study, morphine abuse may adversely affect male fertility potential via a decrease in sperm parameters, especially progressive motility, as well as a decrease in chromatin/DNA quality. It should be noted that additional cellular and molecular studies are needed to know the extent of damages of morphine abuse on spermatozoa and other reproductive indices.
